# Exploring Contact Toxicity of Essential Oils against *Sitophilus zeamais* through a Meta-Analysis Approach

**DOI:** 10.3390/plants11223070

**Published:** 2022-11-13

**Authors:** Fernanda Achimón, Maria L. Peschiutta, Vanessa D. Brito, Magalí Beato, Romina P. Pizzolitto, Julio A. Zygadlo, María P. Zunino

**Affiliations:** 1 Instituto Multidisciplinario de Biología Vegetal (IMBIV), Consejo Nacional de Investigaciones Científicas y Técnicas (CONICET), Av. Vélez Sarsfield 1611, Córdoba X5016GCA, Argentina; 2 Instituto de Ciencia y Tecnología de los Alimentos (ICTA), Facultad de Ciencias Exactas, Físicas y Naturales (FCEFyN), Universidad Nacional de Córdoba (UNC), Av. Vélez Sarsfield 1611, Córdoba X5016GCA, Argentina; 3 Facultad de Ciencias Agropecuarias, Departamento de Recursos Naturales, Cátedra de Microbiología Agrícola, Universidad Nacional de Córdoba, Av. Ing. Agr. Félix Aldo Marrone 735, Córdoba X5016GCA, Argentina; 4 Facultad de Ciencias Exactas, Físicas y Naturales, Departamento de Química, Cátedra de Química Orgánica, Universidad Nacional de Córdoba, Av. Vélez Sarsfield 1611, Córdoba X5016GCA, Argentina

**Keywords:** *Sitophilus zeamais*, essential oils, insecticidal effect, topical application, indirect contact with filter paper, indirect contact with maize

## Abstract

*Sitophilus zeamais* is a primary pest of maize. Our aim was to perform a qualitative review and meta-analyses with 56 scientific articles published from 1 January 2000 to 1 October 2022 dealing with direct (topical application) and indirect (impregnation of essential oils, EOs, onto filter paper or maize grains) contact toxicity of EOs against *S. zeamais*. Three independent meta-analyses of single means of LD_50_ (direct contact) and LC_50_ (indirect contact) were conducted using a random effect model. Essential oils more frequently evaluated were those belonging to Asteraceae, Apiaceae, Lamiaceae, Myrtaceae, Piperaceae, and Rutaceae. The LC_50_ global mean values were 33.19 µg/insect (CI_95_ 29.81–36.95) for topical application; 0.40 µL/cm^2^ (CI_95_ 0.25–0.65) for filter paper indirect contact; and 0.50 µL/g maize (CI_95_ 0.27–0.90) for maize grains indirect contact. The species *Carum carvi*, *Salvia umbratica*, *Ilicium difengpi*, *Periploca sepium*, *Cephalotaxus sinensis*, *Murraya exotica*, *Rhododendron anthopogonoides*, *Ruta graveolens*, *Eucalyptus viminalis*, *Ocotea odorifera*, *Eucalyptus globulus*, *Eucalyptus dunnii*, *Anethum graveolens*, *Ilicium verum*, *Cryptocarya alba*, *Azadirachta indica*, *Chenopodium ambrosioides*, *Cupressus semperivens*, *Schinus molle*, *Piper hispidinervum*, *Mentha longifolia*, and *Croton pulegiodorus* showed LC_50_ or LD_50_ values lower than the global means, indicating good insecticidal properties. Our results showed that EOs have great potential to be used as bioinsecticides against *S. zeamais.*

## 1. Introduction

Maize is one of the most widely grown crops globally serving both as human food and animal feed. According to FAO [1], the average 2017–2019 global production of maize was 1137 million mt [2]. However, despite this great production, maize yield is highly affected by several pests during growth and storage stages. The maize weevil, *Sitophilus zeamais* Motschulsky (Coleoptera: Curculionidae), is a primary field and store pest of maize, producing huge post-harvest losses worldwide. Adults and larvae feed on undamaged grains, causing weight losses of about 40% of the total production [3]. Different synthetic insecticides are currently used to control maize weevil populations, and despite the efficacy of these chemical substances, their repeated application is associated with hazardous effects on living organisms and the environment [4]. In this context, certain natural compounds, such as plant essential oils (EOs), have been proposed as environmentally friendly alternatives to conventional insecticides. Essential oils are complex mixtures of volatile organic compounds (VOCs) that can be extracted from different parts of aromatic plants. Terpenoids and their oxygenated derivatives, alcoholic compounds, phenylpropanoids, aldehydes, esters, epoxides, and ketones are among the major groups of VOCs that are frequently found in the chemical profile of EOs [5]. The insecticidal activity of EOs against different agricultural pests have been widely documented [6,7,8,9].

The efficiency of a given EO and/or their pure VOCs might depend on the mode of penetration into the insect body [10,11,12]. For example, fumigant insecticides enter the insect body through the respiratory system by inhalation; contact insecticides are usually applied to a surface, exerting its effect when insects move through treated areas, or topically on the insect surface; while a stomach insecticide exerts its toxic effect when ingested through the mouth. Most scientific articles dealing with contact toxicity of EOs against *S. zeamais* employ these application methods as follows: EOs applied topically in the insect dorsal surface (direct contact), EOs applied on a filter paper (indirect contact), and EOs applied on maize grains (indirect contact). Even though EOs applied on maize grains would take effect upon ingestion, in practice it is considered a method of indirect contact since insects walk on maize during infestation. The amount of research regarding contact toxicity of EOs against *S. zeamais* is outstanding; however, there is no scientific article that gathers all the evidence using a quantitative method for literature reviews. In this context, meta-analysis is a statistical method used to integrate multiple results from independent primary studies to obtain an unbiased assessment of the available evidence [13].

According to the aforementioned, the aim of the present study was to conduct a systematic review and meta-analyses on the direct and indirect contact toxicity of EOs against the maize weevil to determine trends of insecticidal activities within plant families.

## 2. Results

### 2.1. Scientific Articles Selection

**Figure 1:**
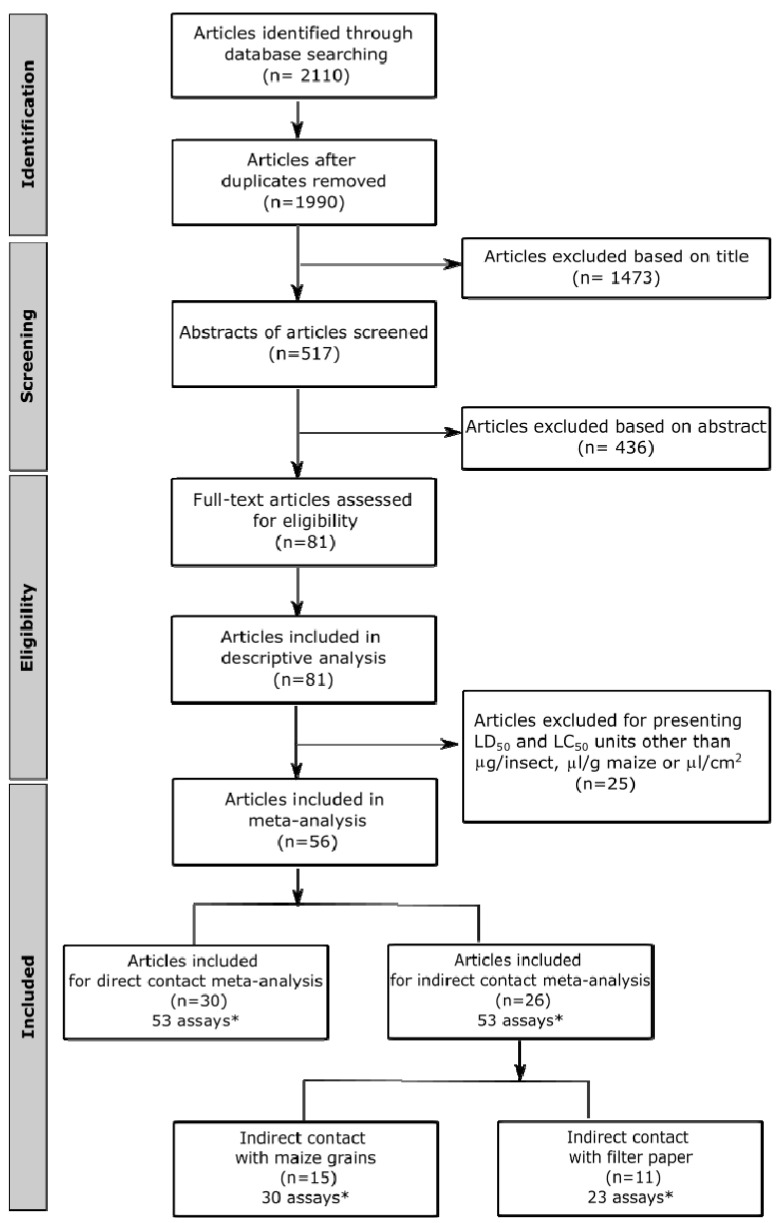
PRISMA flowchart of systematic review and meta-analyses of the excluded and included studies. The flow diagram shows the search results and selection procedure. * Each study used for the meta-analysis was defined for a given plant essential oil (EO)/exposure time/LC50 or LD50 combination.

**Figure 2:**
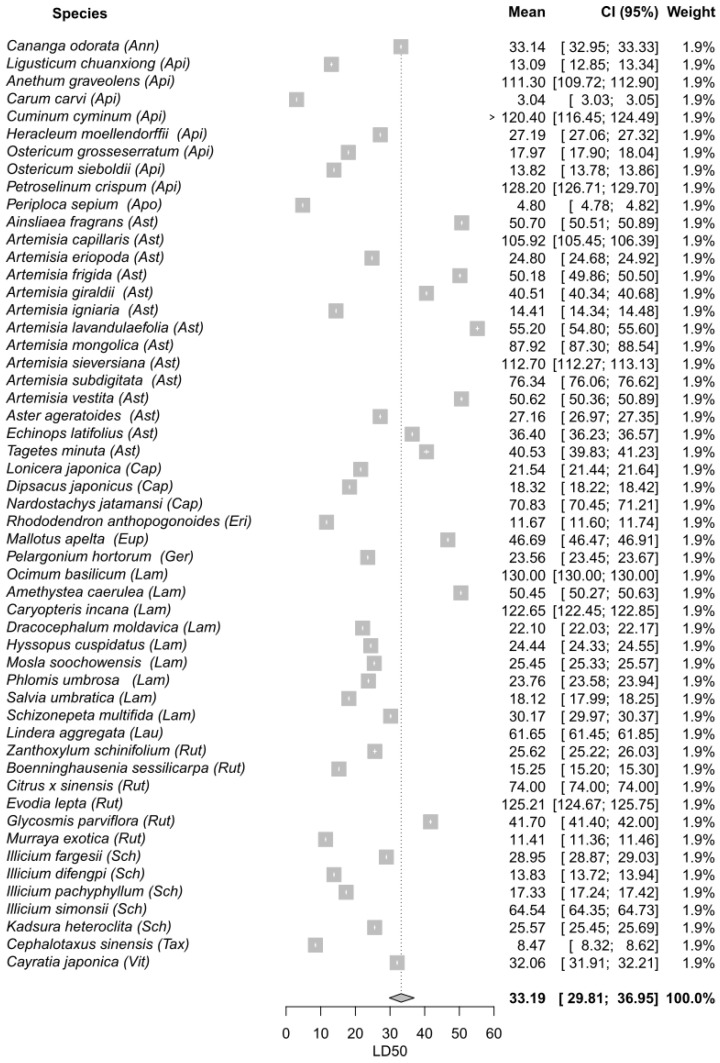
Meta-analysis on the contact toxicity of essential oils (EOs )against *S. zeamais* through topical application. Single LD_50_ means and the LD_50_ global mean are represented along with their corresponding 95% confidence intervals. The abbreviations are as follows: Annonaceae (Ann), Apiaceae (Api), Apocynaceae (Apo), Asteraceae (Ast), Caprifoliaceae (Cap), Ericaceae (Eri), Euphorbiaceae (Eup), Geraniaceae (Ger), Lamiaceae (Lam), Lauraceae (Lau), Rutaceae (Rut), Schisandraceae (Sch), Taxaceae (Tax), Vitaceae (Vit).

**Figure 3:**
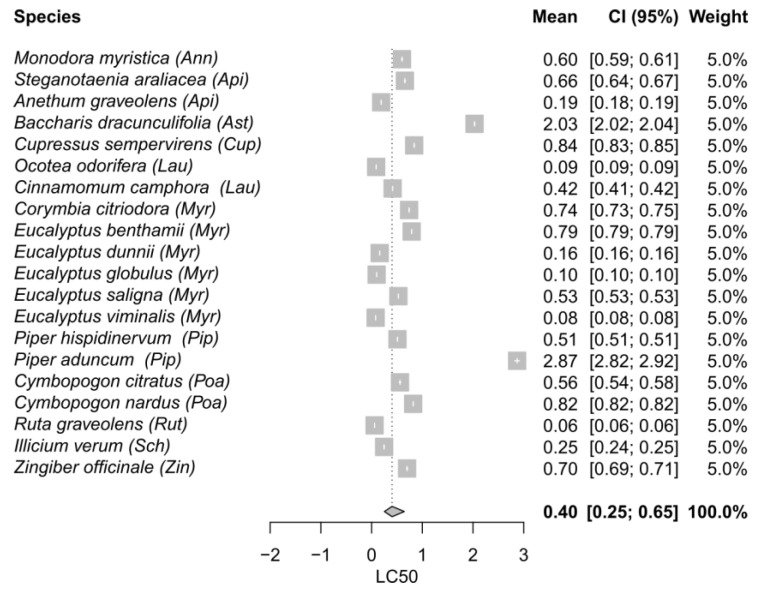
Meta-analysis on the contact toxicity of EOs against *S. zeamais* through filter paper impregnation. Single LC_50_ means and the LC_50_ global mean are represented along with their corresponding 95% confidence intervals. The abbreviations are as follows: Annonaceae (Ann), Apiaceae (Api), Asteraceae (Ast), Cupressaceae (Cup), Lauraceae (Lau), Myrtaceae (Myr), Piperaceae (Pip), Poaceae (Poa), Rutaceae (Rut), Schisandraceae (Sch), Zingiberaceae (Zin).

**Figure 4:**
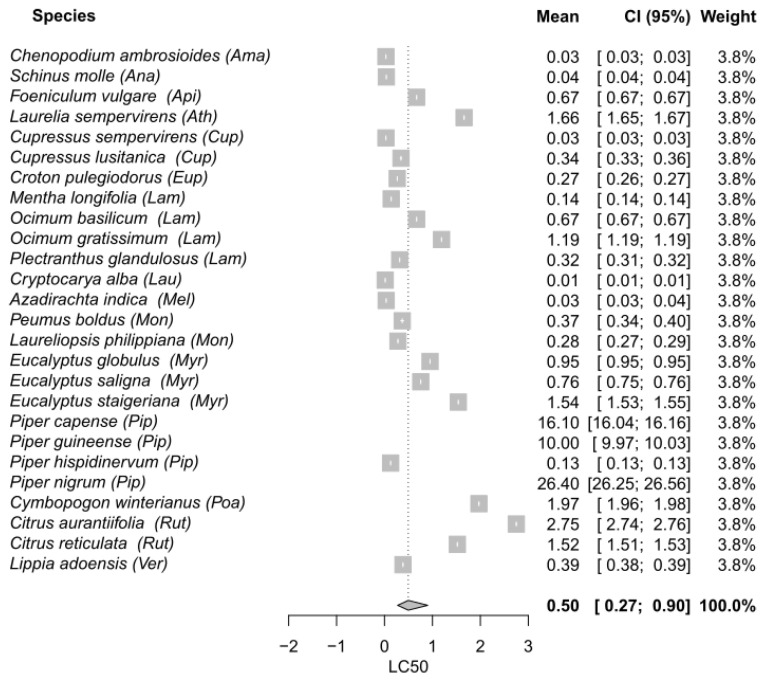
Meta-analysis on the contact toxicity of EOs against *S. zeamais* through maize grains impregnation. Single LC_50_ means and the LC_50_ global mean are represented along with their corresponding 95% confidence intervals. The abbreviations are as follows: Amaranthaceae (Ama), Anacardiaceae (Ana), Apiaceae (Api), Atherospermataceae (Ath), Cupressaceae (Cup), Euphorbiaceae (Eup), Lamiaceae (Lam), Lauraceae (Lau), Meliaceae (Mel), Monimiaceae (Mon), Myrtaceae (Myr), Piperaceae (Pip), Poaceae (Poa), Rutaceae (Rut), Verbenaceae (Ver).

The process of selecting and reviewing articles using the PRISMA flow diagram is shown in Figure 1. A total of 2110 articles were recovered from the database searching. After removing duplicates, 1990 articles were screened based on their titles, which resulted in 1473 records being excluded. The remaining 517 articles were screened based on their abstracts, and 436 were deleted for evaluating toxicity through non-contact methodologies. Finally, an output of 81 articles were retrieved and reviewed for the descriptive revision. Finally, 56 articles (106 assays) attained the inclusion criteria and were included in the meta-analyses (Figure 2, Figure 3 and Figure 4). Out of the 56 final articles, 30 assessed EO toxicity through topical application (direct contact), while 26 evaluated EO toxicity through indirect contact: 15 and 11 records for maize grains and filter paper, respectively.

### 2.2. Descriptive Analysis

Data from the qualitative analysis showed that 46% of scientific articles evaluated insecticidal effect using topical application; conversely, studies on indirect contact were less represented, with 28% and 26% of the total articles for indirect contact using maize grains and filter paper as substrates, respectively. In addition, 65% of topical application articles extracted EOs from the aerial parts, followed by fruits and roots, with 17% and 14% of the total studies, respectively. Regarding maize grain articles, 60% used aerial parts to obtain the EOs, followed by fruits with 18% and seeds with 3%. Similarly, 77% of filter paper articles evaluated EOs obtained from aerial parts, followed by seeds and fruits, with 8% and 4%, respectively. Lower values were obtained for the remaining plant parts within each methodology (data not shown).

Regarding families, 19% of the studies dealing with topical application were conducted using plant species from the family Asteraceae, followed by Apiaceae with 15%, Lamiaceae with 13%, and Rutaceae with 11% of the studies. Within Asteraceae, most studies were conducted using *Artemisia* spp. EOs. In fact, 14% of all the studies that used topical application were conducted with EOs from *Artemisia* spp., followed by *Ilicium* spp. and *Ostericum* spp., with 6% and 4% of the total studies, respectively. Concerning the indirect contact with maize grains methodology, 13% of the studies were carried out with plant species belonging to the family Myrtaceae, followed by Asteraceae, Lamiaceae, and Piperaceae, with 11% of the studies each. Assays performed with EOs from *Eucalyptus* spp. and *Piper* spp. accounted for the majority of studies reported for Myrtaceae and Piperaceae, with 11% of the studies for each genus. Finally, plant species belonging to Myrtaceae, Rutaceae, and Apiaceae were the most frequently used in indirect contact toxicity assays with filter paper, with 19%, 13%, and 10% of the studies, respectively. Furthermore, 17% of articles dealing with the toxicity of *Eucalyptus* spp. accounted for the majority of studies of the family Myrtaceae. Similarly, *Citrus* spp., which was evaluated in 4% of the studies, accounted for the high percentage of studies of Rutaceae (data not shown).

### 2.3. Direct Contact Meta-Analysis

A total of 53 plant EOs belonging to 14 different families were evaluated for topical application giving a LD_50_ global mean value of 33.19 µg/insect (CI_95_ 29.81–36.95). As shown in the forest plot (Figure 2), half of the plant species presented mean values that were lower than the global mean. Most species from Apiaceae, Lamiaceae, and Schisandraceae were good insecticides, with mean values that ranged from 3.04 to 27.19 µg/insect for Apiaceae, 18.12 to 25.45 µg/insect for Lamiaceae, and 13.83 to 28.95 µg/insect for Schisandraceae. Within these plant families, the most effective EOs were: *Carum carvi* (3.04 µg/insect; Apiaceae), *Salvia umbratica* (18.12 µg/insect; Lamiaceae), and *Ilicium difengpi* (13.83 µg/insect; Schisandraceae). Additionally, certain EOs belonging to other plant families that were effective against the maize weevil were: *Periploca sepium* (4.80 µg/insect; Apocynaceae), *Cephalotaxus sinensis* (8.47 µg/insect; Taxaceae), *Murraya exotica* (11.41 µg/insect; Rutaceae), and *Rhododendron anthopogonoides* (11.67 µg/insect; Ericaceae).

### 2.4. Indirect Contact Meta-Analysis

Regarding filter paper meta-analysis, only seven plant species belonging to different families were statistically lower than the global mean 0.40 µL/cm^2^ (CI_95_ 0.25–0.65). The most effective EOs were those extracted from *Ruta graveolens* (0.06 µL/cm^2^; Rutaceae), *Eucalyptus viminalis* (0.08 µL/cm^2^; Myrtaceae), *Ocotea odorifera* (0.09 µL/cm^2^; Lauraceae), *Eucalyptus globulus* (0.10 µL/cm^2^; Myrtaceae), *Eucalyptus dunnii* (0.16 µL/cm^2^; Myrtaceae), *Anethum graveolens* (0.19 µL/cm^2^; Apiaceae), and *Ilicium verum* (0.25 µL/cm^2^; Schisandraceae).

Concerning indirect meta-analysis with maize grains, the global mean was 0.50 µL/g maize (CI_95_ 0.27–0.90). The EOs with the highest insecticidal potential were those obtained from *Cryptocarya alba* (0.01 µL/g maize; Lauraceae), *Azadirachta indica* (0.03 µL/g maize; Meliaceae), *Chenopodium ambrosioides* (0.03 µL/g maize; Amaranthaceae), *Cupressus semperivens* (0.03 µL/g maize; Cupressaceae), *Schinus molleÿ* (0.04 µL/g maize; Anacardiaceae), *Piper hispidinervum* (0.13 µL/g maize; Piperaceae), *Mentha longifolia* (0.14 µL/g maize; Lamiaceae), and *Croton pulegiodorus* (0.27 µL/g maize; Euphorbiaceae).

## 3. Discussion

This study presents a systematic review and meta-analyses on the direct and indirect contact toxicity of EOs against the maize weevil. The EO of a single species usually has a different chemical profile (components and/or relative contents), and hence different bioactivities, according to the plant organ from where it was isolated. For example, Wang et al. [14] reported quantitative and qualitative differences in the chemical composition of *Zanthoxylum schinifolium* leaf and fruit EOs, with the latter being more bioactive in direct contact toxicity assays. The present review showed that, in most studies, EOs were extracted from the aerial parts, i.e., leaves and flowers. This was an expected result since it has been stated that EO yield is usually higher in these organs compared to other plant parts, such as roots or seeds, probably due to the higher density of glandular hairs [15,16,17].

The insecticidal effect of EOs or their pure VOCs depends on the method of application used against the insect. As expected, topical application was the most commonly employed test method since it is one of the most accurate ways to ensure the toxic compound to take contact with the insect. In topical application assays, a known volume of an EO is applied directly to the insect’s body; conversely, in the impregnated filter paper method, the EO is applied to a disk of paper and then insects are released onto the paper from where they pick up an uncertain amount of the EO by contact through the tarsi [18]. In both cases, the penetration of the EO occurs through the cuticle to reach the haemolymph that carries the EO to its target organs. Insect cuticle comprises an external hydrophobic thinner layer (epicuticle) and the internal hydrophilic thicker layer where chitin is prevalent (procuticle). The chitin fibres of the procuticle are predominant in hard body insects (such as weevils), playing an important role in the uptake of the EO; however, certain parts of the insect body, such as intersegmental membranes, sites around the setae and sensilia, lack of a developed chitin layer, thus offering less resistance to the diffusion of EOs [19]. This heterogeneity in the structure of the cuticle could explain the differences in the penetration rates, and thus, in the toxic effect when the same EO is applied through both methodologies [20]. As such, a given EO or VOC can act more efficiently when applied topically compared to indirect contact assays, and vice versa. For example, Hai et al. [21] demonstrated that the LD_50_ values obtained when *Lonicera japonica* EO and its major compound estragole were applied topically on *S. zeamais* were five-fold higher compared to the control (pyrethrum), while the application of the EO and estragole through filter paper impregnation produced a LC_50_ value that was only three times higher than the control, evidencing a better response in indirect contact assays. However, an opposite pattern was observed with linalool (another major constituent of *L. japonica*) that showed a LD_50_ that was only three times higher than pyrethrum in topical application assays, while the LC_50_ obtained in assays with filter paper was nine-fold higher, evidencing that linalool acts more efficiently in direct contact assays. These results showed that within an EO, different compounds may penetrate the cuticle more efficiently according to the methodology. As it was stated before, the cuticle can be considered a two layer lipophilic–hydrophilic system with varying thickness according to the body part. In this context, the interaction between the thickness of the hydrophobic and/or hydrophilic portions of the cuticle and the polarity of the pure compounds (octanol-water partition coefficient; Log P) will ultimately determine their rate of penetration. For example, the estragole Log P value is higher than that of linalool, indicating that it is more lipophilic and has higher penetration of the cuticle when the hydrophilic chitinous layer is thin. However, the penetration capacity of the compound is not necessarily reflected in its bioactivity since many other chemical characteristics of the compounds influence their insecticidal properties [22].

Another species that showed important differences in its effectiveness according to the test method was *Anethum graveolens*. The results from the forest plots showed that the single mean of *A. graveolens* EO was half the global mean in filter paper meta-analysis, and three-fold higher than the global mean in topical application meta-analysis, evidencing better insecticidal properties when impregnated on filter paper compared to the topical application. The major constituent of *A. graveolens* EO is the monoterpene ketone carvone, which has been reported as good insecticide against *S. zeamais* in filter paper tests (Table 1; [23]). The authors evaluated ketones with different molecular structures and discovered that ketones with an extra double bond between the alpha and beta carbons (α,β-unsaturated) have an increased polarizability, which is associated with stronger intermolecular attractive forces. Consequently, α,β-unsaturated ketones are expected to bind with many protein, enzymes, or nucleic acid sites, exerting their insecticidal activity. On the other hand, Fouad and da Camara [24] found that *Citrus aurantifolia*, *Citrus reticulata* EOs, and their major constituent, limonene, were more effective in contact toxicity assays with filter paper compared to contact toxicity using maize grains. This was also the case for *Eucalyptus globulus* that showed an LC_50_ value (0.10 µL/cm^2^) statistically lower than the global mean (0.40 µL/cm^2^) in filter paper indirect contact; while the LC_50_ value (0.95 µL/g maize) was statistically higher than the global mean (0.48 µL/g maize) when the EO was applied in maize grains. On the contrary, as it can be seen in the forest plots, the species *Cupressus semperivens*, *Piper hispidinervum*, and *Eucalypus saligna* were more toxic to *S. zeamais* when the EOs were impregnated on maize grains compared to filter paper. Regardless of the EO absorption through tarsi that both methodologies assume, when EOs are applied on maize there might also be an effect of the toxic compounds through ingestion, therefore having a stomach action as well. For example, safrole and dillapiole, the major constituents of *P. hispidinervum* EO, are part of a group of natural compounds known as phenylpropanoids that are characterized by an aromatic phenyl group and a three-carbon propene tail. Previous studies that evaluated the effect of phenylpropanoids on *S. zeamais*, *S. oryzae*, and *Rhyzopertha dominica* suggested that their toxicity may be due to ingestion and digestion of the compounds in the stomach and not by contact and absorption through the cuticle [25,26]. Additionally, *C. semperivens* and *E. saligna* were characterized by high amounts of the monoterpene hydrocarbons α-pinene and 3-carene in their EOs, among other compounds. Langsi et al. [27] reported that the contact toxicity of these compounds applied on maize led to high levels of mortality on *S. zeamais* adults. Even though single compounds may exert their individual effect against the target insects, the bioactivity of a certain EO usually depends on all its components since synergistic, antagonistic, or additive interactions can occur among them. For example, Kouninki et al. [28] studied the toxicity of α-pinene, 3-carene, and terpinen-4-ol (some major constituents of *C. semperivens* EO) and found that each compound alone produced low mortality against *S. zeamais* adults in contact toxicity tests with maize grains, but when these compounds were combined, a synergic effect among them restored the mortality percentage observed for the EO.

In topical application assays, the most frequently used families were Asteraceae, Apiaceae, and Lamiaceae. Even though most studies within Asteraceae were performed using EOs from the genus *Artemisia*, only the species *A. igniaria* and *A. eriopoda* resulted good insecticides, i.e., LD_50_ values that were lower than the global mean (33.19 µg/insect). The chemical profile of these species showed 1,8-cineole, camphor, and germacrene D as major constituents. Previous studies showed strong contact toxicity when the epoxide 1,8-cineole was applied onto the pronotum of *S. zeamais* [58]. Even though other species of *Artemisia* also have these VOCs as major compounds, they showed a low insecticidal effect. The bioactivity of an EO is usually attributed to its major constituents; however, the presence of minor constituents can lead to additive, synergist, or antagonist properties, affecting the bioactivity of the whole EO [11]. On the contrary, most species from Apiaceae showed good insecticidal properties, with markedly different LD_50_ values that ranged from 3.04 µg/insect to 27.19 µg/insect. The EOs of Apiaceae species were characterized by high percentages of structurally different compounds such as the phenylpropenes myristicin and apiole, the oxygenated monoterpenes α-terpineol (monoterpene alcohol) and carvone (monoterpene ketone), and limonene (monoterpene hydrocarbon). These VOCs have been reported as strong insecticides in direct contact assays due to their capacity for inhibiting acetylcholinesterase (AChE), glutathione-S-transferase (GST), and Na+/K+-ATPase channels activities, thereby interfering with the transmission of impulses in the insect nervous system [103]. Regarding the family Lamiaceae, half of the species tested showed a good insecticidal effect, with similar LD_50_ values that ranged from 18.12 µg/insect to 25.45 µg/insect. According to the chromatographic analyses, the major constituents of Lamiaceae EOs were the oxygenated monoterpenes 1,8-cineole (cyclic ether), thymol (phenolic), 4-terpineol (monoterpene alcohol), geranial or citral (monoterpene aldehyde), and β-caryophyllene sesquiterpene hydrocarbon. The latter three compounds also produced a reduction of AChE, GST, and Na+/K+-ATPase channels activities by interacting with their catalytic subunits [103]. Thymol is an oxygenated monoterpene characterized by a free hydroxyl group in the p-cymene skeleton (phenol group). Previous studies determined the insecticidal effect of thymol against *S. zeamais* and reported that it would exert its toxic effect by binding to GABA receptors, or by inhibiting AChE activity with the phenol group being the part of the molecule responsible for its toxic effect [22,104]. Finally, *Illicium* sp. (Schisandraceae) was one the most frequently evaluated genera in the topical application assays, with three out of four species presenting good insecticidal activities and LD_50_ values that ranged from 13.83 µg/insect to 28.95 µg/insect. Some of the main constituents of these EOs were the oxygenated monoterpenes α-terpineol, carvone, linalool, the sesquiterpenes β-caryophyllene and α-eudesmol, and the phenylpropanoid safrole. The insecticidal activity of the aliphatic monoterpene alcohol linalool has been reported against *S. zeamais* and other insect species, and it would be related to the inhibition of AChE and the interaction of the ligand with the receptor [58,105]. Similarly, the topical application of carvone led to a strong contact toxicity against *S. zeamais*, which was associated with an inhibition of AchE activity [34]. The monoterpene alcohols α-terpineol and terpinen-4-ol were reported as strong insecticides against *S. zeamais* and *S. oryzae*, with mortality percentages that were similar to those of the control DDVP after 24 h of exposure [106,107]. Finally, it is worth mentioning the species *Rhododendron anthopogonoides* (Ericaceae) that displayed good insecticidal properties and 4-phenyl-2- butanone as its major compound. 4-Phenyl-2-butanone showed a strong contact toxicity when tested alone against *S. zeamais*, which was comparable to that of the positive control, the pyrethrum extract [61,108].

Concerning indirect contact meta-analysis using filter paper, seven species belonging to different families presented high insecticidal potential, with LC_50_ values that ranged from 0.06 µL/cm^2^ to 0.25 µL/cm^2^, and a global mean value of 0.40 µL/cm^2^. Three out of the seven species belonged to the genus *Eucalyptus* and presented similar amounts of 1,8-cineole and α-pinene in their EOs. Some VOCs that were detected in the EOs of the remaining species were: the ketones camphor, carvone, 2-undecanone, and 2-nonanone, the phenylpropanoids safrole, anethole, estragole, and p-anisaldehyde. Regarding ketones, Herrera et al. [23] evaluated the insecticidal activity of terpene ketones with different molecular structures, including carvone and camphor, with the former being eight times more toxic than the latter. As it was mentioned above, the α,β-unsaturation may be responsible for the higher bioactivity of ketone. The authors determined through a QSAR analysis that the insecticidal activity was primarily explained by the shape of molecules and the branching of the carbon-atom skeleton, (p-menthane structure and the relative position between the ketone and alkyl groups), with aliphatic and bicyclic structures (such as camphor) exhibiting lower bioactivities. Another study reported a strong insecticidal effect of the non-terpenoid alkyl ketone, 2-decanone, and 3-decanone [109]. On the other hand, different phenylpropanoids were detected in the most effective EOs. Zaio et al. [108] studied the contact toxicity of different phenylpropanoids and found that estragole was four times more bioactive than its positional isomer, anethole. The authors hypothesized that there is a better interaction between the phenylpropanoid and the target molecule when the carbon/carbon double bond is located at the terminal end of the propenyl chain (estragole) than in the middle part of the propenyl chain (anethole). In another study, estragole exhibited a higher toxic effect than anethol on the AChE activity in *S. oryzae* [110]. On the other hand, safrole was reported as a potent contact insecticide against *S. zeamais* and *Tribolium castaneum*. In addition, nutritional experiments revealed that safrole caused an antifeedant effect and a reduction of the insect growth and efficiency of conversion of ingested food through the inhibition of the activity of α-amylase [111].

Regarding the toxicity of EOs through indirect contact (and ingestion) by impregnation of EOs in maize grains, eight species belonging to different families reported the highest insecticidal activities, with LC_50_ values between 0.01 and 0.27 µL/g maize, and a global mean value of 0.50 µL/g maize. The main components of these EOs were: the acids hexadecanoic and oleic acid, the cyclic peroxides ascaridole and isoascaridole, and the monoterpene hydrocarbons cymene, pinene, 3-carene, limonene, sabinene, and phellandrene. Dele [112] studied the contact toxicity of hexadecanoic acid and oleic acid impregnated on maize and found that both organic acids were strong insecticides, with mortality percentages comparable to that of the control pirimiphos-methyl at 24, 48, and 72 h after exposure. On the other hand, the monoterpenoids ascaridole and isoascaridole exhibited strong insecticidal activity against *S. zeamais*. The former has a peroxy group across position 1 to 4, while the latter has two epoxy groups. Accordingly, it would seem that the endoperoxide is important for the toxic effect since ascaridole was reported to be three times more toxic than isoascardole [31]. Regarding monoterpene hydrocarbons, contact toxicity assayed by coating cymene, pinene, and 3-carene onto maize grains showed that these VOCs caused significant mortality on *S. zeamais* and other species of insects [28,113].

## 4. Materials and Methods

### 4.1. Search Strategy

The systematic review and meta-analyses of all peer-reviewed, published studies of contact toxicity of EOs against *S. zeamais* were conducted in accordance with the systematic literature review and meta-analysis reporting guidelines of the Preferred Reporting Items for Systematic Reviews and Meta-Analysis (PRISMA) [114,115]. The search was carried out in four electronic scientific databases: SCOPUS, PubMed, Science Direct, and Google Scholar, and studies published from 1 January 2000 to 1 October 2022 were included. The construct used to compile relevant literature was: “(“*Sitophilus zeamais*”) AND (“essential oils” OR “essential oil”) AND (“LD_50_” OR “LC_50_”) AND (“contact” OR “topical application”)”. The inclusion criteria for the studies were: (1) the studies evaluated the contact toxicity of plant EOs against *S. zeamais* adults; (2) the studies reported data on the concentration killing 50% of the population: lethal concentration, 50% (LC_50_) or lethal dose, 50% (LD_50_); and (3) the studies presented data in µL/cm^2^, µg/insect, or µL/g maize; (4) the studies provided means, sample sizes, and measures of variance (standard error, standard deviation, or confidence interval). When a single paper reported more than one LC_50_ or LD_50_ value due to the use of different populations of *S. zeamais* or the part of the plant from where EO was extracted, data were considered as independent studies.

### 4.2. Study Selection

Reviews, case reports, conference abstracts, letters, and research articles without variables of interest were excluded. Relevant scientific articles were screened by title and abstract to select those that potentially fulfilled the inclusion criteria. The first screening was based on the title, which was considered as an exclusion criterion. For example, if the article dealt with other insect species or evaluated insecticidal activities of natural compounds other than EOs, such as natural formulations, plant extracts, or plant powders, the paper was discarded. However, if any of the keywords were present in the title, the article was included, and the abstract was analyzed to search for relevant information. After selection, full-text papers were thoroughly read, and data were extracted from the selected papers by one reviewer and checked for accuracy by a second reviewer.

### 4.3. Statistical Analyses

The extracted data were harmonized into a Microsoft Excel^®^ spreadsheet and included: first authors, year of publication, plant species, plant family, part of the plant from where the EO was extracted, major compounds of the EO, LC_50_, or LD_50_ value, method of application (topical, filter paper, or maize grains). Data analysis was performed in R software. The input for each study comprised the study size (N), the standardized means, and the standard deviations. Three independent meta-analyses were conducted: one for direct contact through topical application, one for indirect contact through filter paper impregnation, and one for indirect contact through maize grain impregnation. The meta-analyses were conducted through a random effect model. The “metamean” function in the meta package [116] of R software (version 3.6.1) (R Core Team 2016) was used to conduct the meta-analyses of single means and calculate the global LD_50_ and LC_50_ means with their corresponding 95% confidence interval [117]. Three forest plots displaying the single means of each study (LC_50_ value in indirect contact meta-analyses and LD_50_ value in direct contact meta-analysis) with 95% confidence interval limits (CI), the inverse variance study weights, and the global mean with their corresponding 95% confidence interval limits (CI) were generated. Comparisons among single means of EOs and between single means of EOs and global means were carried out to determine the effectiveness of EOs in controlling the maize weevil. Single means were considered as statistically different from the global mean when their confidence interval limits did not overlap. When the means of independent studies were statistically lower than the global mean, the EOs were considered as good insecticides against *S. zeamais*. When two or more articles evaluated the same EO, an average of the LC_50_ or LD_50_ values was calculated for that species before conducting the meta-analyses.

## 5. Conclusions

The penetration of insecticides through the cuticle is one of the most important mechanisms due to the large proportion of insect cuticle surface. The present study explored the insecticidal effect of EOs against the maize weevil through different types of contact methodologies. Still, it should be considered that the different routes of penetration are not always clearly demarcated. For example, since EOs are constituted of volatile compounds, the contact toxicity test results in a combination of both contact and fumigant toxicity. Similarly, toxicity through ingestion of maize grains impregnated with EOs also represents a contact method. However, regardless of the application method, the meta-analyses conducted in the present study showed that EOs have great potential to be used as bioinsecticides against *S. zeamais*. The species *Carum carvi*, *Salvia umbratica*, *Ilicium difengpi*, *Periploca sepium*, *Cephalotaxus sinensis*, *Murraya exotica*, *Rhododendron anthopogonoides*, *Ruta graveolens*, *Eucalyptus viminalis*, *Ocotea odorifera*, *Eucalyptus globulus*, *Eucalyptus dunnii*, *Anethum graveolens*, *Ilicium verum*, *Cryptocarya alba*, *Azadirachta indica*, *Chenopodium ambrosioides*, *Cupressus semperivens*, *Schinus molle*, *Piper hispidinervum*, *Mentha longifolia*, and *Croton pulegiodorus* showed LC_50_ or LD_50_ values lower than the global means, indicating good insecticidal properties. Certain monoterpene ketones (carvone, camphor), monoterpene alcohols (terpinen-4-ol, linalool, thymol, α-terpineol), phenylpropanoids (anethole, estragole, safrole), and monoterpene and sesquiterpene hydrocarbons (pinene, 3-carene, limonene, β-caryophyllene), epoxides and peroxides (1,8-cineole and ascaridole, respectively) were some of the major VOCs shared by these EOs. In this context, recognizing which VOCs or group of VOCs are behind their bioactivity; which VOC functional groups or molecular features enhance their toxicity; how the uptake of the VOC should occur in order to be more effective; and which are their modes of action (e.g., sodium channels, calcium channels, AChE activity, GABA channels, and octopamine receptors) are important aspects to be considered in the study of EOs. These aspects are crucial to develop insecticidal formulations of VOCs that have different target sites, thus reducing the risk of developing resistance and representing a safer alternative to currently used contact insecticides.

## Figures and Tables

**Table 1:** Main components of the plant EOs evaluated for their direct and indirect contact toxicity against *S. zeamais.*

Family	Plant species	EO main components (%)	Ref.
Amaranthaceae	*Cananga odorata*	linalool (21.1), linalool acetate (16.1), α-pinene (12.7)	[29]
	*Chenopodium ambriosioides*	(Z)-ascaridole (29.7), isoascaridole (13.0), ρ-cymene (12.7)	[30,31]
Anacardiaceae	*Schinus molle*	β-pinene (15.4), α-phellandrene (14.9), ϱ-cimene (10.8)	[32]
Annonaceae	*Monodora myristica*	p-cymene (31.5), α-phellandrene (18.1), α-pinene (6.1)	[33]
Apiaceae	*Anethum graveolens*	(S)-carvone (66.4), β-phellandrene+limonene (24.7)	[34]
	*Carum carvi*	(R)-carvone (37.9), limonene (26.5), α-pinene (5.2)	[35]
	*Cuminum cyminum*	cuminaldehyde (39.4), γ-terpinene (15.8), β-pinene (12.4)	[34]
	*Foeniculum vulgare*	limonene (41.8), (E)-anethole (17.9), α-pinene (11.1)	[36]
	*Heracleum moelledorffii*	apiol (11.0), β-pinene (9.2), α-terpineol (7.5)	[37]
	*Ligusticum chuanxiong*	3-butylidenephthalide (20.6), Z-ligustilide (19.6), 4-terpineol (8.8)	[38]
	*Ostericum grosseserratum*	limonene (16.2), 4-terpineol (13.5), myristicin (11.3)	[39]
	*Ostericum sieboldii*	myristicin (30.3), α-terpineol (9.9), α-cadinol (7.2)	[40]
	*Petroselinum crispum*	myristicin (31.5), α-pinene (16.2), apiole (15.9)	[34]
	*Steganotaenia araliacea*	α-pinene, α-copaene	[41]
Apocynaceae	*Periploca sepium*	2-hydroxy-4-methoxy-benzaldehyde (78.8), linalool (2.8), α-terpineol (2.7)	[42]
Asteraceae	*Ainsliaea fragrans*	myristicin (41.3), elemicine (11.9), cis-isosafrole (11.5), borneol (9.1)	[43]
	*Artemisia capillaris*	β-pinene (12.6), germacrene D (8.3), γ-terpinene (8.1)	[44]
	*Artemisia eriopoda*	germacrene D (21.6),	[45]
	*Artemisia frigida*	cis-p-menth-2-en-1-ol (20.8), 1,8-cineole (14.2)1,8-cineole (12.0)	[46]
	*Artemisia giraldii*	β-pinene (13.1), isoelemicin (10.0), germacrene D (5.6)	[47]
	*Artemisia igniaria*	1,8-cineole (14.3), camphor (13.3), germacrene D (8.7)	[48]
	*Artemisia lavandulaefolia*	caryophyllene (15.5), β-thujone (13.8), 1,8-cineole (13.1)	[49]
	*Artemisia mongolica*	1,8-cineole (13.7), germacrene D (10.4), camphor (8.5)	[44]
	*Artemisia sieversiana*	1,8-cineole (9.2), geranyl butyrate (9.2), borneol (7.9), camphor (7.9)	[49]
	*Artemisia subdigitata*	1,8-cineole (12.2), α-curcumene (10.7), β-pinene (7.3)	[47]
	*Artemisia vestita*	grandisol (40.2), 1,8-cineole (14.8), camphor (11.3)	[50]
	*Aster ageratoides*	α-terpineol (10.8), β-caryophyllene (10.3), linalool (7.2)	[51]
	*Baccharis dracunculifolia*	β-pinene (18.0), ledol (13.6), spathulenol (13.4)	[52,53]
	*Echinops latifolius*	1,8-cineole (19.6), (Z)-β-ocimene (18.4), β-pinene (15.5)	[54]
	*Tagetes minuta*	tagetone (11.8), dihydrotagetone (10.7), ocimene (8.8)	[55,56]
Atherospermataceae	*Laurelia sempervirens*	safrole (64.7), methyl eugenol (14.6), 1,8-cineole (1.4)	[57]
Caprifoliaceae	*Dipsacus japonicus*	linalool (11.7), (E)-geraniol (8.5), 1,8-cineole (7.9), β-caryophyllene (5.5)	[58]
	*Lonicera japonica*	estragole (80.1), linalool (6.0), germacrene D (3.1)	[21]
	*Nardostachys jatamansi*	calerene (25.9), patchoulol (10.6), α-gurjunene (7.5)	[59]
Cupressaceae	*Cupressus lusitanica*	umbellulone (18.4), α-pinene (10.0), sabinene (8.2)	[60]
	*Cupressus sempervirens*	α-pinene (17.6), 3-carene (25.9), limonene (10.5), sabinene (9.4), terpinen-4-ol (4.7)	[30]
Ericaceae	*Rhododendron anthopogonoides*	4-phenyl-2-butanone (27.2), nerolidol (8.0), 1,4-cineole (7.8)	[61]
Euphorbiaceae	*Croton pulegiodorus*	p-cymene (23.1), ascaridole (22.5), α-terpinene (9.3)	[62,63]
	*Mallotus apelta*	β-eudesmol (18.6), β-caryophyllene (9.8), β-selinene (6.5)	[64]
Geraniaceae	*Pelargonium hortorum*	1,8-cineole (23.0), α-terpineol (13.2), α-pinene (8.1)	[65]
Lamiaceae	*Amethystea caerulea*	morrilol (25.1), 4-vinylguaiacol (14.3), 2-acetoanisole (14.3)	[66]
	*Caryopteris incana*	estragole (24.8), linalool (14.0), 1,8-cineole (5.2)	[67]
	*Dracocephalum moldavica*	1,8-cineole (31.2), 4-terpineol (22.8), cumyl alcohol (4.2)	[68]
	*Hyssopus cuspidatus*	thymol (19.6), (E)-pinocamphone (15.3), γ-terpinene (14.6)	[69]
	*Mentha longifolia*	1-8-cineole (25.5), menthone (17.9), pulegone (29.9)	[70]
	*Mosla soochowensis*	β-caryophyllene (12.8), spathulenol (6.3), β-eudesmol (6.2)	[71]
	*Ocimum basilicum*	linalool (62.5), methyl chavicol (30.9)	[36,72]
	*Ocimum gratissimum*	(E)-anethole (35.0), limonene (15.6), eugenol (9.1)	[36]
	*Phlomis umbrosa*	geranial (16.5), linalool (13.3), cis-geraniol (7.4)	[73]
	*Plectranthus glandulosus*	cis-piperitone oxide (18.5), fenchone (18.3), piperitone epoxide (17.7), terpinolene (8.7), piperitone oxide (8.9)	[74]
	*Salvia umbratica*	1,8-cineole (16.7), β-caryophyllene (8.4), α-thujone (7.8)	[75]
	*Schizonepeta multifida*	menthone (40.3), pulegone (26.9)	[76]
Lauraceae	*Cinnamomum camphora*	camphor (68.0), linalool (9.0)	[77]
	*Cryptocarya alba*	(E)-β-bergamotene (15.6), viridiflorol (8.5), germacrene-D (7.7)	[78]
	*Lindera aggregata*	α-longifolene (15.1), bornyl acetate (11.4), α-eudesmol (9.1)	[79]
	*Ocotea odorifera*	camphor (43.0), safrole (42.0), spathulenol (2.0)	[80]
Meliaceae	*Azadirachta indica*	hexadecanoic acid (34.0), oleic acid (15.7)	[81,82]
Monimiaceae	*Laureliopsis philippiana*	safrole (39.6), linalool (34.5), 1,8-cineole (8.3)	[83]
	*Peumus boldus*	ascaridole (24.4), 1,8-cineole (14.9), trans-β-ocimene (12.9)	[83]
Myrtaceae	*Corymbia citriodora*	citronellal (61.8), isopulegol (15.5), β-citronelol (7.9)	[84]
	*Eucalyptus benthamii*	α-pinene (54.0), viridiflorol (17.0) 1,8-cineole (10.0)	[85]
	*Eucalyptus dunni*	1,8-cineole (53.5), α-pinene (21.5), viridiflorol (8.3)	[85]
	*Eucalyptus globulus*	1,8-cineole (77.5), α-pinene (14), α-terpineol (1.3)	[85]
	*Eucalyptus saligna*	1,8-cineole (45.2), p-cymene (34.4) α-pinene (12.8)	[85]
	*Eucalyptus staigeriana*	limonene (28.7), geranial (15.2), neral (12.2)	[36]
	*Eucalyptus viminalis*	1,8-cineole (77.0), α-pinene (15.0), viridiflorol (2.3)	[85]
Piperaceae	*Piper aduncum*	dilapiol (74.0), safrol (3.9), sarisan (2.8)	[86]
	*Piper capense*	β-pinene (59.3), sabinene (14.7), α-pinene (10.5)	[87]
	*Piper guineense*	β-caryophyllene (20.8), limonene (15.8), β-pinene (12.1)	[87]
	*Piper hispidinervum*	dilapiol (74.0), safrol (3.9), sarisan (2.8)	[86]
		safrole (82.07)	[36]
	*Piper nigrum*	3-carene (18.5), limonene (14.7), β-caryophyllene (12.8)	[87]
Poaceae	*Cymbopogon citratus*	geranial (40.1), neral (29.7) and myrcene (11.3)	[33]
	*Cymbopogon nardus*	citronellal (36.5), geraniol (25.6), elemol (8.2)	[84]
	*Cymbopogon winterianus*	citronellal (35.5), geraniol (21.8), citronellol (10.9)	[36]
Rutaceae	*Boenninghausenia sessilicarpa*	α-cadinol (12.0), carvacrol (8.8), germacrene D (6.2), o-cymene (6.1)	[88]
	*Citrus aurantiifolia*	limonene (38.9), R-mentha-2,4(8)-diene (5.7), α-terpineol (5.2)	[24]
	*Citrus reticulata*	limonene (80.2), myrcene (6.7), linalool (3.7)	[24]
	*Citrus x sinensis*	limonene (96.1), β-myrcene (1.9), linalool (0.9) α -pinene (0.5)	[89,90]
	*Evodia lepta*	α-pinene (26.6), borneol (7.2), trans-pinocarveol (6.8)	[91]
	*Glycosmis parviflora*	(Z)-caryophyllene (20.6), methyl isoeugenol (11.1), (Z)-β-ocimene (8.9)	[92]
	*Murraya exotica*	spathulenol (17.7), α-pinene (13.3), caryophyllene oxide (8.6)	[93]
	*Ruta graveolens*	2-undecanone (30.7), 2-nonanone (20.8), 4-hydoroxypyridine1-oxide (6.7)	[94]
	*Zanthoxylum schinifolium*	linalool (12.9), α-tumerone (8.9), limonene (6.5), elixene (5.4)	[14]
Schisandraceae	*Illicium difengpi*	safrole (23.6), linalool (12.9), germacrene D (5.3)	[95]
	*Illicium fargesii*	α-terpineol (11.4), carvone (10.9), limonene (9.8)	[96]
	*Illicium pachyphyllum*	trans-p-mentha-1(7),8-dien-2-ol (24.5), limonene (9.7)	[97]
	*Illicium simonsii*	β-caryophyllene (10.3), δ-cadinene (9.5), methyl eugenol (8.9)	[98]
	*Illicium verum*	anethole (77.4), estragole (5.8), p-anisaldehyde (5.6)	[90]
	*Kadsura heteroclita*	α-eudesmol (17.5), 4-terpineol (9.7), δ-cadinene (9.2)	[99]
Taxaceae	*Cephalotaxus sinensis*	α-pinene (38.0) β-caryophyllene (17.0) germacrene D (11.0)	[100]
Vataceae	*Cayratia japonica*	linalool (19.4), trans-α-ionene (11.4), α-terpineol (7.9)	[101]
Verbenaceae	*Lippia adoensis*	geraniol (37.2), linalool (27.7), β-farnesene (10.8)	[102]
Zingiberaceae	*Zingiber officinale*	α-zingiberene (28.9), β-sesquiphellandrene (13.1), Z-γ-bisabolene (12.5)	[33]

## Data Availability

Not applicable.
